# Unraveling the Physicochemical, Nutritional and Antioxidant Properties of the Honey Produced from the *Fallopia japonica* Plant

**DOI:** 10.3390/foods13131959

**Published:** 2024-06-21

**Authors:** Alexandra-Antonia Cucu, Otilia Bobiș, Victorița Bonta, Adela Ramona Moise, Claudia Pașca, Mihaiela Cornea-Cipcigan, Rodica Mărgăoan, Ștefan Dezsi, Sara Botezan, Ecaterina-Daniela Baciu, Alexandru-Ioan Giurgiu, Anamaria Mălinaș, Daniel Severus Dezmirean

**Affiliations:** 1 Faculty of Animal Science and Biotechnology, University of Agricultural Sciences and Veterinary Medicine Cluj Napoca, 400372 Cluj-Napoca, Romania; antonia.cucu@usamvcluj.ro (A.-A.C.); victorita.bonta@usamvcluj.ro (V.B.); adela.moise@usamvcluj.ro (A.R.M.); claudia.pasca@usamvcluj.ro (C.P.); sara.botezan@student.usamvcluj.ro (S.B.); daniela.baciu@usamvcluj.ro (E.-D.B.); alexandru.giurgiu@usamvcluj.ro (A.-I.G.); ddezmirean@usamvcluj.ro (D.S.D.); 2 Department of Horticulture and Landscaping, Faculty of Horticulture, University of Agricultural Sciences and Veterinary Medicine, 400372 Cluj-Napoca, Romania; mihaiela.cornea@usamvcluj.ro; 3 Department of Animal Production and Food Safety, Faculty of Veterinary Medicine, University of Agricultural Sciences and Veterinary Medicine, 400372 Cluj-Napoca, Romania; rodica.margaoan@usamvcluj.ro; 4 Faculty of Geography, Babeş-Bolyai University, 400084 Cluj-Napoca, Romania; stefan.dezsi@ubbcluj.ro; 5 Department of Engineering and Environmental Protection, Faculty of Agriculture, University of Agricultural Sciences and Veterinary Medicine, 400372 Cluj-Napoca, Romania; anamaria.malinas@usamvcluj.ro

**Keywords:** *Fallopia japonica* honey, antioxidant capacity, nutritional benefits

## Abstract

*Fallopia japonica* (FJ), commonly known as Japanese knotweed, is now recognized as one of the most invasive plants in Europe and globally. Despite its widespread presence in Europe and its significant nectar production, there is currently limited scientific data on the unique unifloral honey derived from it. This study examines the physicochemical composition of *Fallopia japonica* honey (FJH) samples collected from various regions in Romania. Additionally, the nutritional and antioxidant profiles of FJH were assessed. The sensory analysis revealed a honey with a brown-caramel color and an intense flavor, characterized by fine, consistent crystals during crystallization. The results indicated that FJH has a high carbohydrate content (fructose: 35.12–40.65 g/100 g; glucose: 28.06–37.79 g/100 g); elevated electrical conductivity (387–692 µS/cm), diastase activity (9.11–17.01 DN), and acidity (21.61–42.89 meq/kg); and substantial total phenolic (89.87–120.08 mg/100 g) and flavonoid (18.13–39.38 mg/g) contents. These findings highlight FJH’s favorable nutritional properties, aligning with the standard codex for honey. The antioxidant profile of FJH demonstrated strong DPPH and ferric reduction antioxidant power (FRAP) activities, comparable to those of buckwheat honey, underscoring its potential health benefits and commercial value. These results provide new insights into how this invasive plant can be harnessed as a valuable resource for sustainable beekeeping practices.

## 1. Introduction

In recent years, there has been a growing focus on health-supportive and functional foods, with honey being one of the most remarkable products used since ancient times for its nutritional value and apitherapeutic properties [1].

It is defined as a natural sweetener, created by honeybees from plant nectar or insect secretions feeding on living plants organs [2]. Being recognized as an easily accessible energy source, honey is acknowledged for its distinct chemical composition, including essential macro- and micro-elements, along with phenolic compounds that offer beneficial effects to human health [3,4]. The authenticity of honey is related to its composition and quality—features strongly influenced by other attributes such as the source of nectar or pollen, climate conditions, soil composition or even post-harvest handling practices [5]. Regarding the latter, assessing the physicochemical attributes of honey and ensuring its quality are essential tasks for beekeepers, as the process enables them to fulfill the international requirements regarding the legal standards for honey authenticity and safe consumption [6].

Based on botanical origin, there are two discernible categories of floral honeys: the unifloral variety, deriving from a predominant species and multifloral honeys, which are comprised of a mixture of various botanical sources, with none predominating [7]. Over the last few decades, unifloral honeys have captured consumer interest due to the possibility that these honeys comprise the unique properties of the plants from which they originate [8] and consequently act as therapeutic antioxidants, reducing the incidence of human diseases [9]. Even though Europe hosts over 100 botanical species capable of yielding ample nectar for unifloral honey production, only a few honey types have been thoroughly documented and defined for their unifloral nature [10].

In this regard, *Fallopia japonica* (FJ), commonly known as Japanese knotweed, *Reynoutria japonica* or *Polygonum cuspidatum*, is an understudied plant with excellent nectariferous potential [11]. Originating from Asia and regarded as one of the most widely dispersed invasive plants globally, causing adverse effects on ecosystems and biodiversity [12,13], FJ is defined as a tall herbaceous perennial species. It can reach heights of up to 3 m [14] and spreads rapidly because of its shared rhizome structure [15]. This rapidly proliferating plant stands out for its robust environmental resilience [16]; thus, eradicating it is exceedingly challenging and costly [17].

Consequently, using this plant for its positive uses can bring forth the potential of a circular economy in which existing biodiversity challenges and the agricultural productivity of a specific territory can be harmonized [18].

It is widely recognized that plants harbor substantial stores of biologically active compounds, functioning as natural antioxidants with nutritional, functional, and health-enhancing attributes [19]. This is also applicable to FJ, as recently, numerous scientific studies have focused on characterizing the chemical and biological properties of this invasive plant [20,21,22,23,24], widely recognized for its utilization in Chinese alternative medicine [25].

Although *FaJ* is recognized as one of the most invasive plants worldwide—particularly in riparian habitats near rivers and waterways, as in the case of Romania [26], with a large contribution to pollination services through its nectar-rich inflorescences [11,27,28] and production of over 200 kg/ha of honey [29]—there is a limited amount of research on *Fallopia japonica* honey (FJH). Only four studies [26,30,31,32] have been conducted, all in Romania, but none of them fully characterize this unique type of honey.

Belonging to the same *Polygonaceae* family, FJH has been compared to buckwheat honey [31,33], sharing the same dark color, particular flavor and high nutritional value [30]. Nevertheless, this variety of honey remains unknown by the majority of consumers, thus lacking sufficient commercial value. Additionally, the absence of relevant analytical data regarding FJH makes it extremely difficult to label it as unifloral honey or to differentiate it from other multifloral or unifloral honey varieties, emphasizing the importance of conducting a comprehensive characterization of this particular honey type.

While there has been extensive study on the pharmacological properties of the *Fallopia japonica* plant, research on its associated honey is notably scarce.

In light of the aforementioned statements, our study represents a pioneering effort to characterize this distinct type of honey, which remains relatively unknown to many beekeepers due to the plant’s late blooming period and invasive tendencies.

Therefore, the present study aims to evaluate the physicochemical composition of FJH across diverse geographical parts of Romania, notably its nutritional profile, to measure its potential as a functional food. Moreover, the antioxidant potential of FJH will also be assessed, proving that the bioactive properties and therapeutic effects of this plant can be transferred to honey, turning the latter into a vehicle for delivering these health-boosting characteristics. In addition, our study aims to enhance our understanding of the distinctive properties of FJH, thereby contributing to the valorization of this type of honey and providing future directions for utilizing this invasive plant through its distinctive honey, mitigating the adverse effects of the FJ plant.

## 2. Materials and Methods

### 2.1. Experimental Sites and Samples

The experimental sites for this study and the gathering and preparing of the samples are the same as in our previous article [26], as the study is a continuation of the research conducted by our team, focused on the FJ plant and honey and the therapeutic effects. Briefly, the study was conducted in the northwestern and western part of Romania, namely three different areas from the (1) Merișor area, Maramureș County (47°39′25.2″ N 23°24′06.7″ E), in the hydrographic confluence area of the Lapus and Somes rivers and Baia Mare depression; (2) the Valea Vinului area, Satu Mare County (47°43′46.9″ N 23°10′26.3″ E), on the Someș river meadow; and (3) the Bocsig area, Arad County (46°25′50.4″ N 21°57′47.3″ E), on the Crișul Alb river meadow [26].

The areas from which the honey was collected are highly dominated by the FJ plant, Merișor being the most affected by its invasiveness, while Bocsig area was the less-impacted experimental site from the point of view of the FJ habitat [26].

The analyses for honey samples were performed at the Centre for Advanced Research and Extension in Apiculture (APHIS-DIA) in Cluj-Napoca, Romania. The denomination used for the honey samples is as follows: FHH1-3 (the samples from Merișor area, Maramureș County), FJH4-6 (the samples from Valea Vinului area, Satu Mare County) and FJH7-9 (the samples from Bocsig area, Arad County).

**Figure 1:**
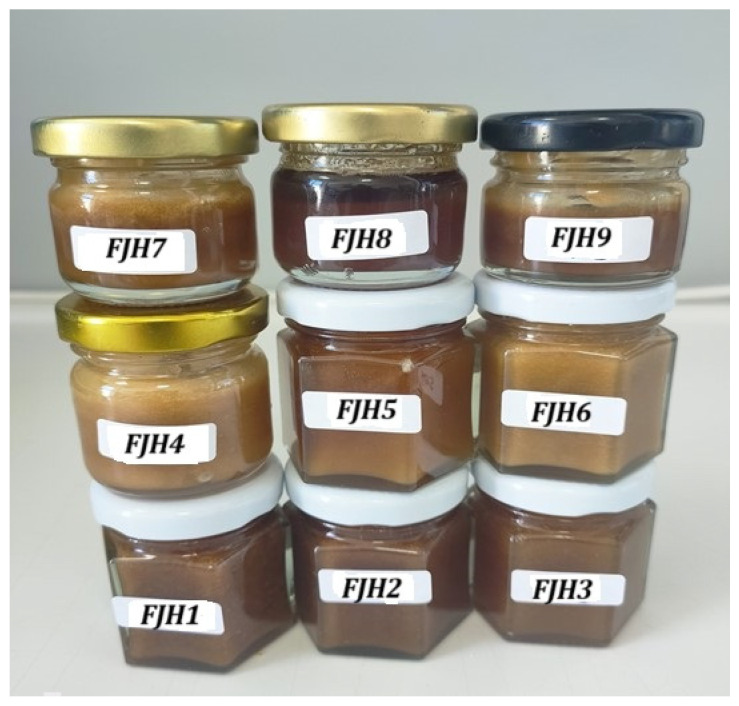
FJH samples gathered from the three experimental sites. (Source: Alexandra-Antonia Cucu personal collection).

From the sites mentioned above, we were able to find a limited number of certified beekeepers who were engaged in the production and commercialization of FJH. The gathering and processing the samples were conducted in line with our previous study [26]: nine samples of FJH (three samples from each location and different apiaries) were obtained directly from local beekeepers in November 2022, two months after the honey was produced (September 2022). These samples were meticulously preserved in hermetic glass containers and maintained at a temperature of 5 °C until the analyses were conducted (Figure 1).

### 2.2. Chemicals

All chemicals used in the analysis were of analytical-grade purity.

Gallic acid and quercetin were procured from Karl–Roth (Karlsruhe, Germany). Additionally, chemicals such as Folin–Ciocâlteu reagent, FeSO_4_, 2,2-diphenyl-1-picrylhydrazyl (DPPH), 2,4,6-tri(2-pyridyl)-s-triazine (TPTZ), 6-hydroxy-2,5,7,8-tetramethylchroman-2-carboxylic acid (Trolox), and FeCl_3_ were acquired from Sigma–Aldrich (St. Louis, MO, USA). High-quality analytical-grade solvents and buffers were employed for both HPLC and spectrophotometric analyses. Deionized water with a conductivity of 0.067 S/cm was generated using a Millipore Milli-Q device.

### 2.3. Botanical Origin Identification

The melissopalynological analysis followed the method outlined by Louveaux et al. [34]. Acetolysis was omitted from the procedure. Samples of 10 g of finely homogenized honey underwent dissolution in 20 mL of H_2_SO_4_ (5 mL/L). This mixture was then subjected to centrifugation at 4500× *g* for 15 min. The resulting supernatant was discarded, and the process was repeated using bi-distilled water to ensure the complete removal of honey sugars. The remaining pellet was affixed to a slide using Kaiser’s glycerol gelatine (Merck KGaA, Dramstadt, Germany) with the optional addition of a few drops of fuchsin, and the palynological examination followed. Slide examination was conducted using an Olympus CX41 optical microscope. One thousand (1000) total pollen grains were counted for the presentation of frequencies as percentages.

### 2.4. Sensory Analysis

To evaluate the sensory parameters of FJH, namely the appearance, consistency, color, smell and taste, we employed the method used by Dobrinaș et al. [35], respecting the Romanian national standard SR 784-3:2009 regarding honey quality requirements for producers, sale and analysis methods [36]. Briefly, the samples underwent homogenization through gentle mixing with a glass rod, followed by filtration and subsequent settling until completely clear.

The appearance was determined by observing each honey sample jar (Figure 1), the consistency was assessed by using a wooden spatula, and the color was established visually in daylight against a white background. The aroma and taste were evaluated through smelling and tasting the sample directly. The sensory evaluation was carried out by a panel comprising untrained individuals, both male and female, aged between 19 and 22 years. Panelists were recruited from the students at the University of Agricultural Sciences and Veterinary Medicine of Cluj-Napoca, Romania, and they signed a written informed consent regarding their willingness to participate to the study. The testing was performed at the APHIS-DIA laboratory on two different days, and the participants were not able to share information.

After the evaluation of each FJH sample, each participant filled in a questionnaire, where they had to choose between different characteristics of each analyzed parameter. For appearance, the participants had to choose between clean, homogeneous, with impurities, without impurities or inaccurate; for consistency, they chose between fluid, fluid-viscous, viscous, fine crystallized and crystallized; for color, they chose between dark, brown, brown-caramel, and reddish-brown; for smell, they chose between vegetal, conifer resins, and fine floral smell; and for taste, they chose between sweet, medium-sweet, bitter, astringent, and metallic.

### 2.5. Physicochemical Analysis

The physicochemical parameters of honey were performed in triplicate, following the standardized protocols outlined by the International Honey Commission’s (IHC) Harmonised Methods [37] and in agreement with the European standards imposed through Codex Alimentarius [38] and the EU Honey Directive [39].

#### 2.5.1. Sugar Profile Content

For the determination of sugar profiles, we followed the method used by Pașca et al. [40] involving HPLC (high-performance liquid chromatography) analysis of carbohydrates, using a customized Alltima Amino 100 stainless steel column (manufactured by Alltech, Nicholasville, KY, USA) and a Shimadzu Liquid Chromatograph LC-10 AD (Shimadzu Corporation, Kyoto, Japan), equipped with a degasser, dual pumps, an autosampler, a temperature-controlled oven, a controller, and a refractive index detector to separate sugars through chromatography. The calculation relied on calibration curves generated from standard solutions spanning various concentrations (0.5–80 mg/mL), with regression coefficients (r^2^) that exceeded 0.998. A 5% honey solution was used for the determination, following the method protocol. Quantification involved comparing the peak area obtained with that of standard sugars. The results were then expressed as g per 100 g of honey.

Regarding the quantification of total carbohydrates and energetic value, the total amount of carbohydrates was calculated as follows: Total carbohydrates = 100 − (g salt + g proteins + g lipids), while the formula used to determine the energy value for FJH was that proposed by Merrill and Watt (1995) [41]:

Energy (kcal) = 4.1 × (g protein + g carbohydrates) + 9.3 × (g fat)(1)

#### 2.5.2. Moisture Content

Moisture content was determined using a method adapted from the IHC [37], which involved measuring the refractive index of a liquid honey sample using an Abbe refractometer (Abbé WAY-S Selecta—Spain). The refractive index measured at 20 °C on the refractometer was subsequently converted to water content using a standard table. Results are expressed as a percentage of water.

#### 2.5.3. Electrical Conductivity

Electrical conductivity involves the conductometric determination of the aqueous solution of FJH samples of an established concentration. The method was used from the harmonized methods of the IHC (2009) [37], using a Hanna Instruments Conductometer. A solution representing 20% dry matter of honey was used in the determination.

#### 2.5.4. Diastase

A photometric method adapted by Pașca et al. [40] using a spectrophotometer UV-1900i (Shimadzu, Japan) was employed in assessing the diastase activity of the honey samples (4% solution, according to the employed method). The diastase activity was calculated using the following formula: 20 × DO590, where DO590 represents the absorbance. Results are expressed in Schade units per gram of honey.

#### 2.5.5. Hydroxymethylfurfural (HMF)

Following the recommendations of the IHC, the concentration of HMF content was analyzed using the method of high-performance liquid chromatography with photodiode array detection (HPLC-PDA) [37], employing a Shimadzu VP system (Kyoto, Japan), for a 20% honey solution. The operational parameters of the chromatographic system were previously reported by Pașca et al. [40]. The concentration of HMF from the honey samples was established following the indications of the IHC [37].

#### 2.5.6. Acidity and pH Content

The total acidity of the FJH samples was calculated as the sum of free acidity—assessed by neutralization with a sodium hydroxide solution—and lactonic acidity—determined by titration with excess sodium hydroxide, followed by neutralization curve analysis of the excess sodium hydroxide using sulfuric acid. The results were expressed as milliequivalents of sodium hydroxide per kilogram of honey (meqNaOH/kg honey).

The method has been adapted for use with automatic titration in the APHIS-DIA Laboratory [40].

The FJH pH determination was carried out using an automatic titrator (Titroline Easy, SCHOTT, Mainz, Germany) with a solution prepared from 10% (*w*/*v*) honey.

#### 2.5.7. Protein Content

The protein profile of the honey samples was obtained by using the Bradford method, which entails the reaction between the Bradford reagent and the proteins found in the analyzed honey samples. The method used was that described by Azeredo et al. [42], with the modifications of Paşca et al. [40]. The calibration curve was created using a BSA (bovine serum albumin) standard solution, applying concentrations from 5 µL/mL to 250 µL/mL, while the absorbance of the determined proteins was measured at 595 nm on a spectrophotometer UV-1900i (Shimadzu, Japan), using a 1:1 honey solution.

The results are expressed as a percentage, after multiplying the read value from the calibration curve by the dilution factor 2 and then dividing by 10.

#### 2.5.8. Lipid Content

The method used to determine the lipid content was the Soxhlet method [43]. Briefly, 2 g of homogenized sample was weighed onto a filter paper, which was wrapped and inserted into the paper cartridge. The clean and dry extraction beakers, together with the boiling stones, were weighed, and the paper cartridge containing the sample and solvent (70 mL n-hexane) was fixed in the extraction beakers of the Soxhlet apparatus (Soxtherm, Gerhardt, Germany). The extraction temperature was 140 °C, the extraction time was 3 h 25 min, the washing time was 30 min and the evaporation of the solvent in a hot-air current was carried out for 10 min. After the extraction, the glasses were placed in the oven at 60 °C for two hours, then left in the desiccator to reach the constant temperature. The cups were weighed, and the final result was expressed as a percentage based on the following formula:

Total lipids (%) = (Pf − Pi)/m sample × 100,

where Pf = final cup mass, Pi = initial cup mass, and m sample = sample mass.

### 2.6. Total Phenol Content

The total phenolic content was assessed using the classic Folin–Ciocâlteu method, which has been modified over the years by various researchers [44,45] and adapted in our research for a BioTek Synergy 2 multichannel spectrophotometer (BioTek Instruments, Winooski, VT, USA). Briefly, 25 μL of the honey sample (20% (*w*/*v*) solution), along with 125 μL of 0.2N Folin–Ciocâlteu reagent, was dispensed into microplate wells. After a 5 min incubation period, 100 μL of 75 g/L Na_2_CO_3_ was added. The mixture was allowed to stand in the dark at room temperature for 2 h, and then the absorbance was measured at 760 nm. A calibration curve was constructed using various concentrations (0.005–0.25 mg/mL) of gallic acid (y = 7.2769x + 0.0625, r^2^ = 0.999). The results were expressed as gallic acid equivalents (GAE) in mg/100 g of honey.

### 2.7. Total Flavonoid Content

For FJH samples, the method employed was that previously used by Mărghitaș et al. [46]. The absorbance was read for all samples (20% (*w*/*v*) solution) at 415 nm, and the quantification was performed using a calibration curve made from different dilutions of quercetin (1 mg/mL) (y = 61.031x − 0.0098, r^2^ = 0.998). The results were expressed in mg quercetin equivalents (QE)/100 g of the sample.

### 2.8. Determination Antioxidant Activity of FJH

The antioxidant activity of FJH was assessed using two methods, namely free radical scavenging activity (DPPH) and the ferric reduction antioxidant power (FRAP).

The 2,2-Diphenyl-1-picrylhydrazyl (DPPH) method used for FJH samples was that described by Brand-Williams et al. [47] and modified by Meda et al. [48]: 0.75 mL of honey solution (0.1 mg/mL) in deionized water was combined with 1.5 mL of 0.03 mg/mL DPPH (Sigma Aldrich GmbH, Steinheim, Germany) in methanol. The mixture was vigorously shaken, then incubated in darkness for 30 min, and the absorbance of the remaining DPPH was determined at 425 nm against a blank.

The findings for the honey samples were presented as % DPPH and determined by calculating the percentage of radical scavenging activity using the following formula:

DPPH % = (A Control − A Sample/A Control) × 100(2)

The concentration that caused 50% scavenging of free radicals is presented as IC_50_. The lower the IC_50_ value, the higher the antioxidant activity.

The FRAP assay was carried out following the method proposed by Benzie et al. [49], with some changes proposed by Aljadi and Kamaruddin (2004) [50]: the FRAP solutions were prepared by combining 2 mL of a 10 mM TPTZ (2,4,6-tripyridyl-s-triazine) solution in 40 mM HCl, 2 mL of 20 mM FeCl_3_, and 20 mL of 0.3 M acetate buffer at a pH of 3.6. To prepare the honey solution, 10 g of honey was dissolved in a 100 mL mixture of water and methanol (1:1 ratio) and filtered through Whatman number 4 filter paper. A calibration curve was established using different concentrations of FeSO_4_ (ranging from 0.1 to 1 mM), and the absorbance of the samples were converted into FRAP values (mM FRAP) by normalizing them against the absorbance of standard solutions multiplied by the FRAP value of 1 mM FeSO_4_. Then, 300 µL of honey solution (0.05 g/mL) was added to 2.7 mL of the FRAP solution, and the absorbance was measured at 593 nm after incubation at 37 °C for 6 min.

### 2.9. Statistical Analysis

The data from the honey samples underwent analysis of variance to reveal any statistically significant differences (*p* < 0.05). Formerly, the Shapiro–Wilk test was performed to assess the normal distribution of the dataset. Additionally, to assess the impact of experimental site origins on the values of physicochemical and bioactivity parameters, a T-test was employed (*p* < 0.05). The statistical analyses were carried out by the use of SPSS software (version 19.0). The data are displayed as means ± standard error. The factoextra package was utilized to perform principal component analysis (PCA). Heatmap and dendrograms (Euclidean distance with complete linkage) were generated to allow for an easier interpretation of the relationships and discrepancies between the honey samples, as well as their collection areas and bioactivities, using the ggplot, cluster R, and dendextend packages from R (version 4.0.5).

## 3. Results and Discussion

### 3.1. Botanical Origin Identification of FJH Samples

The botanical origin of honey is established using the melissopalynological analysis method. In this case, as FJ is an invasive plant species, the pollen spectrum of this unifloral honey type is significantly influenced by its geographical origin. In our case, the honey samples originated from three different areas: (1) the Merișor area, Maramureș County; (2) the Valea Vinului area, Satu Mare County; and (3) the Bocsig area, Arad County. The melissopalyological analysis was carried out for each region separately. Following the palynological analysis, it became evident that the three regions are distinguished by the various pollen types found alongside *FJ* pollen.

Besides the FJ pollen, the samples from the Merișor area, Maramureș County, are rich in Fagaceae—*Castanea sativa* pollen, followed by Asteraceae H (*Helianthus* type), Brassicaceae, and Asteraceae J (*Centaurea jacea* type). When identifying the pollen spectrum of the sample from the Valea Vinului area, Satu Mare County, close to Maramures County, we found that the pollen types that accompany the FJ pollen are Fabaceae—*Trifolium* sp., Asteraceae H, Asteraceae J, and Rosaceae. Both counties have a presence of Asteraceae H and Asteraceae J pollen in common. Instead, the Bocsig area, Arad County, is characterized by the presence of Rosaceae pollen, followed by Brassicaceae, Malvaceae—*Tilia* sp., and Asteraceae J. (family and plant species of the pollen types from the analyzed honey samples, Supplementary Table S1). The sampling region and the vegetation that was present at the time of nectar collection may have had an impact on the variety of pollen types from the FJH samples, as the FJ plant is known to be an invasive species.

Given the lack of scientific literature detailing the pollen spectrum of FJH [30], it is necessary to corroborate the pollen analysis with the physicochemical parameters and sensory parameters of FJH [26,32], in order to obtain a full description of the characteristics of this honey type [51].

The analysis unveiled a diverse array of pollen types from various plant species, spanning across multiple plant species. These pollen grains exhibit geographic specificity, reflecting the unique flora of each region. Despite the honey samples being collected from three distinct geographical areas of Romania during the same period, their compositions differ due to regional variations in plant species. Notably, none of the identified pollen types constituted a majority (>45%) of the sample.

**Figure 2:**
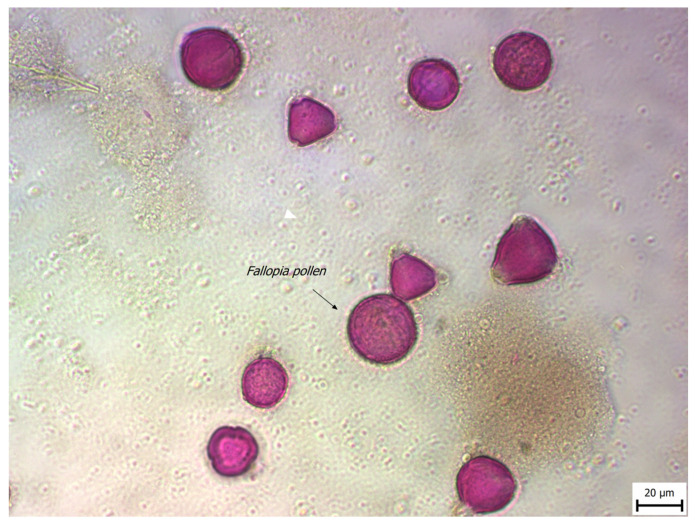
*FJ* pollen type identified in the honey samples.

However, the pollen percentages of various unifloral honey kinds can vary significantly due to the large number of over- or under-represented pollen types. The pollen shape from FJ flower and other types of pollen present in this honey type are visualized in Figure 2. In addition, a comparative analysis with honeys that have similar characteristics in terms electrical conductivity, carbohydrate profile, mineral content, diastase index, HMF content, etc., was also performed. These include buckwheat honey (*Fagopyrum esculentum*)—from the same Polygonaceae family [52,53,54,55,56,57,58,59]—and manuka honey [60].

### 3.2. Sensory Parameters

The sensory parameters established for FJH are presented in Table 1. All samples exhibited a clean appearance, without impurities, while the consistency was from fluid-viscous to fine-crystallized (almost all analyzed samples have a fine crystallization because the fructose/glucose ratio was comprised between 1.08 and 1.15—lower than the ratio of fluid honey and higher than hard crystallized honey). As for the color, FJH has a brown-caramel color with some reddish-brown shades; a general coniferous, floral or vegetal aroma in terms of smell; and a medium sweet taste, with a metallic and refreshing aftertaste of fresh mint. The parameters of FJH are similar to those established for buckwheat honey [56]. The sensory analysis is very important as it can elucidate the product’s attributes and influence consumer choice in buying this type of honey.

### 3.3. Physicochemical Characteristics of FJH from Different Regions of Romania

The findings regarding physicochemical parameters analyzed in the nine FJH samples from three different areas of Romania (Merișor, Valea Vinului and Bocsig) are presented in Table 2.

#### 3.3.1. Sugar Content

The sugar content in honey depends mostly on the botanical and geographical origin of the plant, but other conditions such as climate, processing or storage conditions may also influence this parameter [46]. Generally, honey contains a high concentration of sugars, especially monosaccharides (fructose and glucose), disaccharides (sucrose, turanose, maltose, trehalose) and trisaccharides (melezitosee, erlose). [5]. All these sugars play an important role in determining honey’s energy content and moisture-absorbing or viscosity properties [61].

The present study showed that for the analyzed FJH samples, the level of fructose was the highest of all sugars, ranging from 35.12 g/100 g in the Bocsig area to 40.65 g/100 g in the Valea Vinului area, followed by glucose, where significant differences (*p* < 0.05) can be observed between the Valea Vinului area and the other two experimental sites. From the disaccharides group, turanose exhibited the highest concentration in all honey samples, notably at the Merișor site (2.09 g/100 g). Moreover, FJH samples were characterize by the presence of trisaccharides, the most notable being erlose, with the highest concentrations in the Merişor area (0.65–1.79 g/100 g), followed by Bocsig (0.35–0.81 g/100 g), and the lowest amounts in Valea Vinului, while melezitose was detected only in the honey samples from the Bocsig site (0.81 g/100 g). Overall, significant differences among the areas of collection have been shown, particularly in the case of fructose and sucrose, respectively.

According to our study’s findings on the combined levels of fructose and glucose in all nine FJH samples, the concentrations were above 60%, and sucrose content did not exceed 5%, complying with the EU Council Directive regarding honey standards [39].

As the F/G ratio in FJH samples is above 1, ranging from 1.08 (Valea Vinului site) to 1.15 (Merișor site), we can conclude that this type of honey is generally found in a slow crystallization form [46,62]. Our results are similar to those exhibited by Bobiș et al. on FJH from the western part of Romania [30] but higher than those exhibited for buckwheat and manuka honey in terms of fructose and glucose [60].

#### 3.3.2. Moisture Content

The sugar content is associated with water content as the latter has an important role in the crystallization of honey and also for all the physical and microbiological properties of it [63,64]. Generally, all analyzed FJH samples had an average moisture value lower than 20%, respecting the European legislation regarding the maximum value of moisture content in honey [37,38,65]. However, two samples exceeded the threshold, with values of 21.0% and 20.7 (from Maramureș County and Arad County, respectively). Even if the moisture levels from the samples in our study were higher compared to other honey types (ranging from 17.40% to 21.0%), this can be explained by the fact that, as Thrasyvoulou et al. mentioned, not only *Calluna vulgaris* but also other unifloral honey varieties, including *Polygonum aviculare* (from the same botanical family as *Fallopia japonica*), may exhibit a higher moisture content [51], depending on the nectar sources and weather conditions in the region where the honey is produced [66].

The moisture content values in our study are comparatively higher than those reported by Pătruică et al. (16.76% water content) for knotweed honey [32] or by Nešović et al. for buckwheat honey [67], but they are in agreement with those reported by other authors for other unifloral honey varieties [30,57,62,68].

#### 3.3.3. Electrical Conductivity

Electrical conductivity generally serves as a physicochemical indicator associated with the botanical source of honey, often utilized alongside melissopalynological analysis for distinguishing between honeydew and nectar honey, as well as for categorizing unifloral types [62]. The mean values of electrical conductivity for the FJH samples exhibit lower statistical differences between the Merișor and Valea Vinului experimental sites, while for the Bocsig area, the values were statistically higher—values ranged from 598 µS/cm to 660 µS/cm. These recorded values fall below the maximum threshold of 0.8 mS/cm stipulated for blossom honey in both the *Codex Alimentarius* (2001) and the EU Directive for honey (2002) [38,39]. Comparable results were reported by Bobiș et al. for the same honey variety, whereas for buckwheat honey, the values are generally lower regarding this parameter [67,69].

The electrical conductivity parameter is often related to the minerals found in honey [69]. In a previous study, Cucu et al. [26] evaluated the main macro-, micro- and trace elements found in FJH from the north and northwestern part of Romania, revealing a high content of calcium (Ca), potassium (K) and magnesium K and values of trace elements below 1 mg/kg.

#### 3.3.4. pH Content and Acidity

The pH values did not exceed the threshold of 4.1 for all the analyzed honey samples, with significant differences between the samples and the three experimental sites (*p* < 0.05), particularly for samples collected from Bocsig with the highest value (i.e., 3.95 ± 0.15). Although there is no standard pH value for honey, determining this parameter is crucial as it can affect honey’s stability and freshness [69]. As our study revealed low pH values for all honey samples, it could be stated that FJH inhibits the growth of microorganisms. Our results are consistent with findings from a previous study on FJH [32], eucalyptus honey from Portugal [70] and buckwheat honey from Poland [57].

Honey encompasses a wide range of organic acids that provide its acidity, serving as a defense mechanism against microbial presence [71]. According to EU Council Directive (2001), honey acidity should not exceed 50 meq/kg [65]. This metric serves as an indicator of honey’s freshness and the extent of potential fermentation processes [5]. Various organic acids, along with factors such as geographical origin and harvest season, can impact the acidity levels of honey [72]. In the present research, the acidity levels observed in the analyzed samples varied from 21.61 meq/kg (Bocsig area) to 42.89 meq/kg (Valea Vinului area), below the limit stated above. The variances between the mean values for total acidity were significant (*p* < 0.05) between Valea Vinului and the other two experimental sites but showed no statistical difference between the Merișor area and Bocsig area. The Valea Vinului area had the highest concentration of this parameter (42.89 meq/kg), probably caused by the storage conditions used by the beekeeper. However, these findings indicated the absence of any fermentation in all FJH samples and therefore are in alignment with the standard values indicative of fresh honey. Higher values were reported for Croatian Himalayan honey, with an average of 35.92 meq/kg [6], and lower values were reported for different unifloral and multifloral honeys collected from the Vojvodina region [73].

#### 3.3.5. Hydroxymethylfurfural (HMF) Content

In order to evaluate the freshness parameter of FJH, the HMF and diastase activity were assessed. Various factors have been documented to impact the levels of HMF and diastase activity, including temperature and duration of heating, storage conditions, pH levels or botanical origins [5]. For instance, HMF is a byproduct of fructose degradation, which happens during honey processing and extended storage periods [74]. Thus, it tends to accumulate during heating or aging [75].

In the present research, HMF values of all honey samples were below the maximum threshold of 40 mg/kg, with statistical differences between all analyzed areas (*p* < 0.05). However, the three samples from Satu Mare County (Valea Vinului area) did not exhibit any difference between them. With a maximum value of 5.16 mg/kg (Valea Vinului area) and a minimum value of 0.72 mg/kg (Bocsig area), all the analyzed samples met the international criterion [38,65]. The findings for this physicochemical parameter were quite different to those documented for buckwheat from Poland [57], where the values ranged from 0.30 mg/kg to 7.90 mg/kg, and to those of FJH from Western Romania, where the maximum average value was 9.93 mg/kg [30]. In addition, compared to manuka honey, our results are lower than those reported by Sęk et al. [76].

#### 3.3.6. Diastase Content

Diastase is an enzyme secreted by the salivary and hypopharyngeal glands of worker bees during the maturation process of honey and is regarded as a marker of both honey freshness and its appropriate processing [1]. According to the European regulations, the minimum acceptable diastase activity level in all types of honey (excluding baker’s honey and citrus honeys, which have lower values) is 8, expressed as the diastase number (DN) [65]. The diastase content value ranged from 9.11 DN to 17.01 DN, with the maximum levels logged in the Bocsig area, while the minimum levels were determined for the Merișor area. The significant statistical difference (*p* < 0.05) of the diastase activity between the experimental sites can be explained by the fact that this parameter may be affected by different factors, including the age of the bees, the colony physiological state, the duration of nectar collection, nectar quantity or sugar content [77]. Similar values were reported for FJH by Bobiș et al. (2019) [30]. However, the values reported here are lower than the diastase activity found for Algerian honeys [78], Portuguese honeys [79] or special Croatian honey [6], but they are higher than those noted for the sunflower honey samples of Serbia [63] or raw buckwheat honey from Poland [80] in terms of dark-colored honey types.

According to the data provided in Table 2, it is evident that the initial freshness of FJH is confirmed, as both the HMF content and diastase activity conform to the European regulations.

### 3.4. Nutritional Value of FJH

The nutritional value of FJH is presented in Table 3.

Generally, the nutritional value of honey has been linked with its health properties, as studies have witnessed the positive effect that food in general, and honey in particular, can have in reducing the risk of developing specific diseases or treating them [1]. One of the parameters for assessing the nutritional value of honey is the sugar content, as it represents the main source of energy that it brings when incorporated into the diet [64]. As mentioned before, the honey samples of FJ have a considerable amount of fructose and glucose.

Other components that could influence the nutritional value of honey are the mineral elements that play a crucial role in human biological systems, acting as catalysts in numerous biochemical reactions [81]. Regarding the mineral content present in the same FJH samples, Cucu et al. (2024) demonstrated that this honey variety represents a good source of macro-elements, especially Ca, K and Mg, and also micro- and trace-elements such as Cu (copper), Se (selenium) and Ni (nickel) [26]. These latter elements could have a positive effect on the immune system, bone structure or glucose metabolism, but only if the maximum recommended daily intakes do not surpass 1 mg for Ni and 0.9 mg in the case of Cu [82].

Apart from its main components represented by simple sugars, honey also contains small amounts of other compounds, which have numerous beneficial nutritional effects. One of these classes of compounds are the proteins, present in small amounts in honey, namely 0.1–0.5% [5]. The majority of the protein content varies depending on the type of honey (ranging from 0.2 to 0.4 mg/100 g for blossom honey and 0.4 to 0.7 mg/100 g for honeydew honey) and is considered to be a key factor in the therapeutic properties of honey [66]. The protein contents of the FJH samples had average values ranging from 0.19% (Maramureș County) to 0.38% (Arad County). These values represent nearly half or even less than what other authors have reported for other honey unifloral varieties [60,80,83,84], including Bobiș et al. for other FJH samples from Romania [30].

In our research, the lipid content of FJH was low, with a maximum of 0.14% observed for the Valea Vinului area samples. These data are very different from what was assessed regarding the lipid content for FJH samples from the same part of Romania in a different year (2019) (values ranging between 0.12 and 0.52%) [30]. This could lead us to the conclusion that the lipid content of honeys is also influenced by the geographical and climate conditions of the year when the honey was produced. However, the values reported in other unifloral honey varieties produced in a European Atlantic area were below those covered by our research (with a maximum of 0.02%) in terms of lipid content [84].

Even if the lipid content of FJH is insignificant in terms of its contribution to the energy content of this functional food product, its carbohydrate content, as depicted in Table 3, contrasts in terms of the nutritional value of the honey. FJH showed an ample composition of carbohydrates, with small differences between the mean values of the three analyzed experimental sites (Valea Vinului—81.32%, Bocsig—80.93%, and Merișor—80.93%).

The salt content of honey refers to the concentration of salt dissolved in a liquid medium [70]. In the case of FJH, the salinity showed that the Merișor area had the highest values, while between the Valea Vinului area and Bocsig area, there were no statistical differences (*p* < 0.05). The values of all honey samples were below 0.2%. Higher results for this parameter were described for other unifloral honeys (eucalyptus, chestnut and heather) [70] and polifloral honeys [85].

While our results may not confirm the nutritional importance of FJH in terms of protein and lipid content, the substantial carbohydrate and macro-element (Ca, K and Mg) content in this variety of unifloral honey can still serve as an energy source for those who consume it. The general recommended daily intake of honey for health benefits is typically 50 g to 80 g [86], constituting approximately 12% of the recommended daily energy intake, considering FJH’s reported energy value is around 330 kcal per 100 g.

Moreover, honey is considered a rich source of natural antioxidants, and its potential as a supplement in the human diet by providing a range of antioxidant-rich nutrients is quite evident [87]. As it will be depicted in the following sub-chapter, FJH possesses important amounts of phenolic content, and given that diets abundant in polyphenols are frequently linked to a reduced occurrence of numerous chronic conditions [88], the consumption of FJH as part of one’s dietary routine is recommended.

### 3.5. Antioxidant Capacity of FJH Samples

The total phenolic content (TPC) together with the total flavonoid content (TFC) and antioxidant potential of the nine FJH samples is visualized in Table 4.

The TPC content in the honey samples varied between 89.87 mg GAE/100 g (FJH7) and 120.08 mg GAE/100 g (FJH4). Overall, the honey samples that presented the highest phenolic content originated from the Valea Vinului and Merișor areas. Regarding the total flavonoid content, the values ranged between 18.13 mg CE/100 g (FJH1) and 39.38 mg QE/100 g (FJH6). Significant phenolic and flavonoid content was recorded in honey samples collected from the Valea Vinului area, particularly in the cases of FJH4 and FJH6 samples. However, no significant differences in TPC and TFC among the areas of collection were recorded (*p* < 0.01). Unfortunately, as the studies on FJH are quite scarce, the only two research works that we could use to compare our results were those of Bobiș et al. [30] and Pătruică et al. [32]. These studies have underlined higher levels of TPC, ranging from 100 to 195 mg GAE/100 g for FJH samples collected from the western part of Romania [30] and an average of 195.0 mg GAE/100 g for the samples harvested from the southwestern part of Romania [32]. In the case of TFC, our findings were similar to those reported by Bobiș et al. [30] but higher than those observed by Pătruică et al. [32].

As concerns the antioxidant activity, the two methods used for this evaluation were the DPPH and FRAP assays.

The chemical constituents present in honey, especially the phenolic compounds, play an essential role regarding the antioxidant capacity [66,89], as these antioxidant components in honey are believed to act as dietary supplements against oxidative stress [90]. Nonetheless, numerous studies have assessed the positive correlation between phenolic compounds and the antioxidant capacity of honey [68,91].

As honey’s phenolic content primarily originates from the nectar that bees gather from plants [92], the antioxidant potential of honey is closely tied to the botanical source [93,94]. Moreover, research indicates a correlation between antioxidant capacity and honey color intensity, with darker hues indicating higher antioxidant properties and thus a greater phenolic content [66].

The results from Table 4 showed a medium antioxidant activity, with a percentage of inhibition ranging from 35.41% (IC_50_-14.15) (Bocsig area) to 55.87% (IC_50_-8.88) (Merișor area). The lower percentage of inhibition was attributed to Arad County, while the higher to Maramureș County. All experimental sites exhibited statistical differences between the DPPH inhibition values. Our results are in accordance with those published by Pătruică et al. [32], who observed an average of DPPH antioxidant activity of 57.22%. As for the FRAP assay, the Merișor area showed the highest antioxidant capacity, while between the other two experimental sites, there were no statistical differences (*p* < 0.05).

If compared to buckwheat honey, the antioxidant properties of FJH exhibit lower TFC content and thus a lower antioxidant capacity for the honey samples collected in the USA and Poland, respectively [57,95]. Nonetheless, the study conducted by Puścion-Jakubik et al. [96] showed lower values in DPPH and FRAP than our findings. Moreover, compared to manuka honey, our results had higher values in terms of DPPH activity but lower values in terms of TFC or TPC [97].

### 3.6. Geographical Discrimination and Statistical Analysis

**Figure 3:**
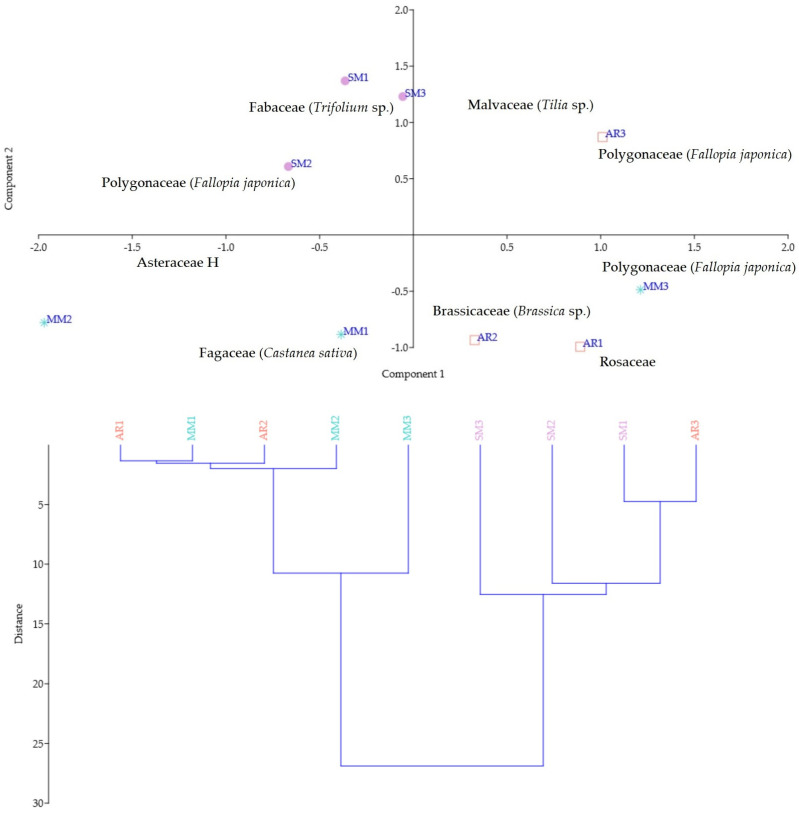
PC (**upper**) and HCA (**lower**) of the honey samples according to their geographic origin and melissopalynological analysis, where blue stands for Maramureș area, orange for Arad area and pink for Satu Mare area.

PC modeling was used to investigate the melissopalinological analysis correlated with the geographical origin of the evaluated honey samples. The projection of the variables describes the correlation between the samples and identified pollen types, whereas the distance from the origin indicates their influence on the variables (Figure 3). Following the quadrants, the samples collected from the Valea Vinului area were grouped due to the secondary pollen (i.e., *Trifolium* sp.) and important pollen types identified (i.e., *Fallopia japonica*). The samples from Valea Vinului were closely followed by AR3 from the Bocsig area, which presents as secondary pollen of the *Fallopia japonica* type. In the following quadrant, sample MM3 was dominant in *Fallopia* sp., *Brassica* sp. and Asteraceae pollen types, which were also detected in FJH7 and FJH8 samples from the Bocsig area. Although in samples MM1 and MM2 FJ pollen was presented as important pollen, Fagaceae (*Castanea sativa*) and Asteraceae H were identified as secondary pollen types.

HCA was computed to better evaluate the samples according to their identified pollen types and their area of collection. The samples from Valea Vinului were grouped with sample FJH9 from Bocsig due to their similarities in the presence of Brassicaceae (*Brassica* sp.), Polygonaceae (*Fallopia japonica*), and Fabaceae (*Trifolium* sp.). Sample FJH3 proved to be slightly different from the other samples collected from the Merișor area due to the presence of FJ pollen as an important pollen type.

Nonetheless, the remaining samples from Bocsig area are rich in Rosaceae and Brassicaceae pollen types and were correlated with the ones from the Merișor area that presented pollen types of Asteraceae H and Fagaceae (*Castanea sativa*). Their relative closeness may be exemplified by their similarities in terms of the identified pollen genera.

To distinguish honey from several distinct geographic regions, certain investigators have implemented a mathematical algorithm to forecast the geographical origin of honey using the frequency of pollen grains from equivalent types of honey samples harvested from different areas. Furthermore, implementing this method in conjunction with statistical clustering and correlation analysis has proven advantageous for distinguishing honey samples of different geographical and botanical origins. This method has been confirmed to be accurate in determining honey authenticity and detecting potential adulteration [98].

**Figure 4:**
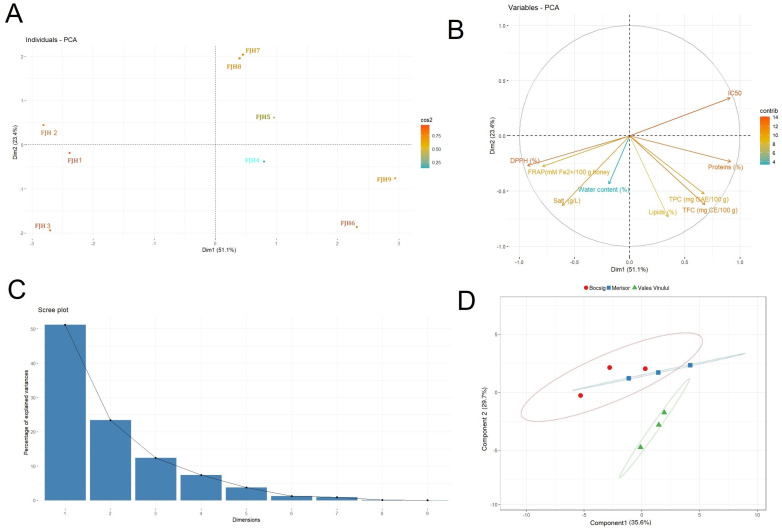
PCA representations of the FJH samples (**A**), and nutritional values, phenolics and antioxidant activities (**B**). A total of 77.2% of the overall variance was attributed to the first two dimensions (**C**). Discriminant analysis (PLS-DA) plot of the physicochemical parameters, and antioxidant activities of the samples according to their areas of collection (**D**). Ellipses reveal the points of each level of the sets of groups and represent 95% confidence intervals. The first axis explained 35.6% and the second 29.7% of the overall variance of the centroids.

According to the second computed PCA, distinct associations among the collection areas of the FJH samples and their nutritional composition, phenolic content and antioxidant activities were assessed using an assortment of statistical approaches (Figure 4A,B). The first dimension explained 51.1% and the second explained 23.4% of the overall variance (Figure 4C, Supplementary Table S2). Following the quadrants, the first two underline the FJH samples collected from the Merișor area that were discriminated from the other collection areas. Sample FJH3 (Merișor) is slightly further positioned from samples FJH1 and FJH2 due to its accumulation in total phenolics and nutritional composition (i.e., particularly salt and water content). Samples FJH1 and FJH2 presented resemblances regarding total phenolic content, carbohydrates and energy value. The following quadrant highlights the FJH samples collected from the area of Bocsig, which presented elevated levels in total phenolic and flavonoid content but also in nutritional composition, mainly regarding the contents in proteins, carbohydrates and energetic value. These samples were proven to be rich sources not only of the FJ pollen type but also of Asteraceae H (i.e., *Helianthus* sp.), which may influence the accumulation of nutritional compounds. Conversely, significantly reduced water content has been recorded in these samples. Sample FJH6 from Valea Vinului was closely grouped with FJH9 from the Bocisg area due to the similarities of both samples in nutritional composition and total phenolic and flavonoid content. Noteworthy, a relatively increased protein content was detected, which may be due to their geographical origin but also their botanical origin (i.e., subsequent to FJ pollen type, predominance in Fagaceae, and Asteraceae). A score plot of the PLS-DA model using the physicochemical dataset and antioxidant activities of the evaluated FJH samples revealed a joint clustering of the samples collected from the regions of Arad and Maramureș (Figure 4D). Notably, the integration of the physicochemical analysis revealed a differentiated discrimination of the FJH samples according to their area of collection, particularly regarding samples collected from Satu Mare situated in different ellipses, suggesting the potential correlation and significance of the parameters included in the computed matrix. This is in line with previous research that highlighted the utilization of physicochemical characteristics to distinguish monofloral and polyfloral honeys [99]. Furthermore, other findings suggest that, in conjunction with floral origin, the geographical provenance of the honey samples is equally significant for the phenolics content and antioxidant activity, even regarding samples originating from the same species [100,101].

**Figure 5:**
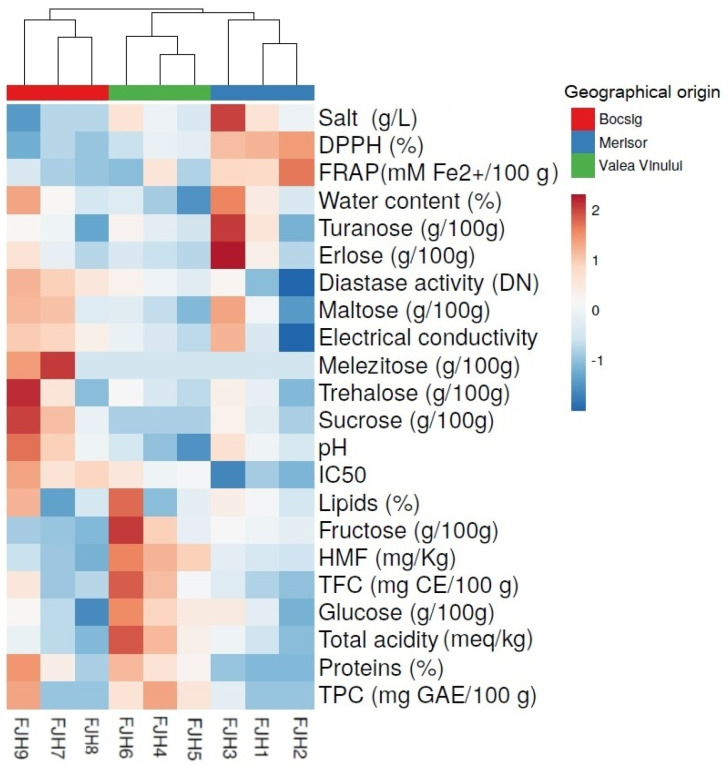
Heatmap and cluster analysis visualization of physicochemical analysis, phenolics and antioxidant activities in FJH samples. Columns indicate the FJH samples according to their collection sites, whereas rows represent the evaluated bioactive compounds, and antioxidant activities. Based on the detected bioactivities, the cells are highlighted accordingly, where blue denotes a significant negative association and red denotes a significant positive association.

The heatmap visualization revealed distinct clusters of honey samples not only based on their physicochemical parameters, phenolic compounds and antioxidant activities but also on their collection areas (Figure 5). The first cluster highlights the grouping of the samples collected from the Bocsig area, which presented elevated levels in melezitose, trehalose, sucrose, and pH particularly in the cases of FJH7 an FJH9 samples. Significant inhibitory levels (IC_50_) were recorded in all samples. Conversely, the samples from the Bocsig area presented relatively lower levels of glucose, and turanose. In the second cluster, the samples from the Valea Vinului area presented similarities and elevated levels in fructose, glucose, HMF, and total acidity, with regard to samples FJH4 and FJH6. In the final cluster, the samples from the Merișor area presented relatively reduced diastaze activity and electrical conductivity, particularly regarding sample FJH2. Furthermore, comparatively lower levels in maltose and trehalose were recorded in FJH1 and FJH2 compared to sample FJH3, which presented significant accumulation of turanose, maltose, and erlose. In contrast, all samples from this area presented significant antioxidant activities, as seen by the positive values according to the importance score.

## 4. Conclusions

This paper presented, for the first time, a comprehensive botanical, sensory and chemical profile of FJH, serving as a vital tool in discerning the authenticity of this specific honey variety. Also, the study offers valuable insights into a unique type of honey that has received limited attention so far and remains relatively unknown to many beekeepers. Further studies are needed to completely characterize the pollen type profile present in FJH. The sensory evaluation indicated a fine crystallization of honey samples, a brown-caramel color with reddish-brown shades, and a medium sweet taste. Moreover, the physicochemical parameters of unifloral FJH showed the distinct characteristics of this honey type, including a high content of sugars (especially fructose and glucose), naturally higher diastase activity, and an acidic pH. All the analyzed samples were in alignment with the standard values indicative of fresh honey. The total polyphenol and flavonoid contents showed high antioxidant activity both for DPPH and FRAP assays. Various statistical methods were utilized to examine the relationships among the collection areas of FJH samples and their nutritional composition, phenolic content, and antioxidant activities. These analyses revealed that FJH samples collected from three distinct regions in Romania shared similarities in terms of pollen type, total phenolic content, antioxidant activity, and carbohydrate and energy values. Moreover, integrating physicochemical analysis highlighted a discernible differentiation among the FJH samples based on their collection area, particularly evident in samples collected from the Valea Vinului area, compared to the other investigated areas (Merișor and Bocsig). Further studies must include the analysis of more samples to acquire a representative picture of the characteristics of FJH to reaffirm the nectariferous potential of the FJ plant so that beekeepers can utilize it as a valuable beekeeping resource.

## Figures and Tables

**Table 1:** Sensory parameters of FJH analyzed samples.

Sample	Appearance	Consistency	Color	Smell	Taste and Persistency
FJH 1	clean, homogeneous, without impurities	fine crystallization	brown-caramel with brown shades	conifer resin, light floral smell	Medium sweet taste and a refreshing aftertaste of fresh mint
FJH 2	clean, homogeneous, without impurities	fine crystallization	brown-caramel with reddish-brown shades	conifer resin, fine floral smell	Medium sweet taste, light metallic and a refreshing aftertaste of fresh mint
FJH 3	clean, homogeneous, without impurities	fine crystallization	brown-caramel with reddish-brown shades	vegetal aroma, conifer resin, fine floral smell	Medium sweet taste, metallic and a refreshing aftertaste of fresh mint
FJH4	clean, homogeneous, without impurities	fine crystallization	brown-caramel	fine floral smell	Medium sweet taste, metallic and a refreshing aftertaste of fresh mint
FJH5	clean, homogeneous, without impurities	fine crystallization	brown-caramel with reddish-brown shades	vegetal aroma, conifer resin	Medium sweet taste, metallic and a refreshing aftertaste of fresh mint
FJH6	clean, homogeneous, without impurities	fine crystallization	brown-caramel	vegetal aroma, conifer resin, fine floral smell	Medium sweet taste, metallic and a refreshing aftertaste of fresh mint
FJH7	clean, homogeneous, without impurities	fine crystallization	brown-caramel	vegetal aroma, conifer resin, fine floral smell	Medium sweet taste, metallic and a refreshing aftertaste of fresh mint
FJH8	clean, homogeneous, without impurities	fluid-viscous	brown-caramel with reddish-brown shades	conifer resin	Medium sweet taste, metallic and a refreshing aftertaste of fresh mint
FJH9	clean, homogeneous, without impurities	fine crystallization	brown-caramel with reddish-brown shades	vegetal aroma, conifer resin	Medium sweet taste, metallic and a refreshing aftertaste of fresh mint

**Table 2:** Physicochemical parameters of FJH samples.

Parameter	FJH1	FJH2	FJH3	Merișor Area	FJH4	FJH5	FJH6	Valea Vinului Area	FJH7	FJH8	FJH9	Bocsig Area
Fructose (g/100 g)	37.05 ± 0.15 ^bc^	36.72 ± 0.69 ^c^	37.26 ± 1.01 ^bc^	37.01 ± 0.27 ^A^	38.68 ± 1.11 ^b^	36.79 ± 1.01 ^c^	40.65 ± 0.91 ^a^	38.70 ± 1.93 ^A^	35.35 ± 0.11 ^d^	35.12 ± 0.13 ^d^	35.53 ± 0.09 ^d^	35.33 ± 0.20 ^B^
Glucose (g/100 g)	32.31 ± 1.45 ^d^	29.39 ± 1.14 ^de^	34.39 ± 1.11 ^c^	32.03 ± 2.51 ^A^	35.79 ± 0.99 ^b^	34.37 ± 0.87 ^c^	37.79 ± 0.66 ^a^	35.98 ± 1.71 ^A^	30.80 ± 1.55 ^d^	28.06 ± 2.01 ^e^	33.45 ± 1.09 ^cd^	30.77 ± 2.69 ^A^
Sucrose (g/100 g)	0.62 ± 0.35 ^d^	nd	1.24 ± 0.09 ^c^	0.62 ± 0.22 ^B^	nd	nd	nd	0.00 ± 0.00	2.12 ± 0.66 ^b^	0.81 ± 1.21 ^d^	3.11 ± 1.09 ^a^	2.01 ± 1.15 ^A^
Turanose (g/100 g)	1.46 ± 0.39 ^b^	0.72 ± 1.1 ^d^	2.09 ± 0.39 ^a^	1.42 ± 0.68 ^A^	1.12 ± 0.09 ^c^	0.99 ± 0.05 ^c^	1.32 ± 0.07 ^b^	1.14 ± 0.16 ^A^	1.21 ± 0.19 ^bc^	0.66 ± 0.95 ^d^	1.29 ± 0.13 ^b^	1.05 ± 0.34 ^A^
Maltose (g/100 g)	1.25 ± 0.31 ^b^	0.68 ± 0.07 ^c^	1.76 ± 1.1 ^a^	1.23 ± 0.14 ^A^	0.98 ± 0.08 ^c^	0.82 ± 0.06 ^c^	1.13 ± 0.03 ^b^	0.97 ± 0.15 ^A^	1.66 ± 0.18 ^a^	1.12 ± 0.11 ^b^	1.68 ± 0.11 ^a^	1.48 ± 0.31 ^A^
Trehalose (g/100 g)	0.29 ± 0.08 ^bc^	0.10 ± 0.03 ^c^	0.39 ± 0.07 ^b^	0.26 ± 0.14 ^A^	0.24 ± 0.04 ^bc^	0.18 ± 0.02 ^c^	0.34 ± 0.03 ^b^	0.25 ± 0.08 ^A^	0.44 ± 0.18 ^b^	0.11 ± 0.05 ^c^	0.76 ± 0.04 ^a^	0.43 ± 0.03 ^A^
Erlose (g/100 g)	0.65 ± 0.52 ^b^	nd	1.79 ± 0.43 ^a^	0.81 ± 0.09 ^A^	0.07 ± 0.06 ^d^	nd	0.21 ± 0.03 ^c^	0.09 ± 0.01 ^A^	0.35 ± 0.23 ^c^	nd	0.81 ± 0.11 ^b^	0.38 ± 0.04 ^A^
Melezitose (g/100 g)	nd	nd	nd	0.00 ± 0.00	nd	nd	nd	0.00 ± 0.00	1.09 ± 0.32 ^a^	nd	0.81 ± 0.22 ^a^	0.63 ± 0.05 ^A^
F/G	1.14 ± 0.04 ^b^	1.24 ± 0.07 ^a^	1.08 ± 0.05 ^c^	1.15 ± 0.08 ^A^	1.08 ± 0.00 ^c^	1.07 ± 0.03 ^c^	1.07 ± 0.01 ^c^	1.07 ± 0.00 ^A^	1.14 ± 0.05 ^b^	1.25 ± 0.03 ^a^	1.06 ± 0.03 ^c^	1.15 ± 0.09 ^A^
Electrical conductivity (µS/cm)	533 ± 84.58 ^c^	387 ± 43.78 ^d^	680 ± 79.34 ^a^	533 ± 46.5 ^A^	533 ± 16.16 ^c^	506 ± 18.8 ^c^	562 ± 20.19 ^c^	533 ± 28.00 ^A^	650 ± 19.21 ^a^	598 ± 25.66 ^b^	660 ± 18.39 ^a^	636.00 ± 33.29 ^A^
pH	3.8 ± 0.05 ^b^	3.73 ± 0.08 ^b^	3.92 ± 0.04 ^a^	3.81 ± 0.09 ^AB^	3.63 ± 0.05 ^bc^	3.54 ± 0.09 ^c^	3.72 ± 0.08 ^b^	3.63 ± 0.09 ^B^	3.97 ± 0.08 ^a^	3.8 ± 0.07 ^b^	4.1 ± 0.04 ^a^	3.95 ± 0.15 ^A^
Total acidity (meq/kg)	25.36 ± 2.13 ^c^	22.13 ± 1.98 ^d^	29.51 ± 2.11 ^bc^	25.66 ± 3.69 ^B^	37.58 ± 3.23 ^b^	31.7 ± 4.03 ^b^	42.89 ± 3.33 ^a^	37.39 ± 5.59 ^A^	24.56 ± 2.05 ^c^	21.61 ± 2.01 ^d^	28.69 ± 2.05 ^bc^	24.95 ± 3.55 ^B^
HMF (mg/Kg)	1.88 ± 0.15 ^b^	1.70 ± 0.19 ^bc^	2.22 ± 0.11 ^b^	1.93 ± 0.26 ^B^	4.55 ± 0.30 ^a^	4.12 ± 0.28 ^a^	5.16 ± 0.35 ^a^	4.61 ± 0.52 ^A^	1.16 ± 0.25 ^d^	0.72 ± 0.35 ^d^	1.62 ± 0.33 ^c^	1.16 ± 0.45 ^B^
Diastase activity (DN)	11.44 ± 1.58 ^d^	9.11 ± 2.14 ^d^	14.58 ± 3.00 ^c^	11.71 ± 2.74 ^B^	14.04 ± 0.40 ^c^	13.35 ± 0.28 ^c^	14.77 ± 0.42 ^c^	14.05 ± 0.71 ^B^	16.32 ± 0.40 ^a^	15.42 ± 0.56 ^b^	17.01 ± 0.55 ^a^	16.25 ± 0.79 ^A^

Different superscript lowercase letters within each row indicate statistically significant differences between the samples at a confidence level of *p* < 0.05. Different superscript uppercase letters denote significant differences among the areas of collection. Reported results represent the mean ± standard deviation values obtained from three replicates (*n* = 3); nd—non detected.

**Table 3:** Nutritional value of FJH.

Samples/	Water Content (%)	Proteins (%)	Lipids (%)	Salt (%)	Total Carbohydrates (%)	Energy Value (kcal/100 g)
Parameters						
FJH1	19.63 ± 1.15 ^b^	0.19 ± 0.00 ^b^	0.08 ± 0.01 ^b^	0.16 ± 0.03 ^b^	79.91 ± 0.19 ^bc^	329.38 ± 4.80 ^b^
FJH2	18.71 ± 1.04 ^c^	0.19 ± 0.00 ^b^	0.06 ± 0.00 ^bc^	0.14 ± 0.02 ^b^	78.54 ± 0.21 ^c^	323.42 ± 4.44 ^c^
FJH3	21.00 ± 1.11 ^a^	0.20 ± 0.00 ^b^	0.09 ± 0.03 ^b^	0.20 ± 0.01 ^a^	80.93 ± 0.22 ^b^	332.93 ± 4.76 ^ab^
Merișor area	19.77 ± 1.15 ^A^	0.19 ± 0.00 ^A^	0.07 ± 0.01 ^A^	0.16 ± 0.03 ^A^	79.79 ± 1.19 ^A^	328.57 ± 4.80 ^A^
FJH4	18.17 ± 0.70 ^c^	0.32 ± 0.03 ^a^	0.04 ± 0.01 ^c^	0.14 ± 0.01 ^b^	81.32 ± 0.74 ^ab^	335.3 ± 2.80 ^a^
FJH5	17.40 ± 0.56 ^c^	0.29 ± 0.01 ^b^	0.07 ± 0.00 ^b^	0.13 ± 0.01 ^b^	80.67 ± 0.82 ^b^	332.7 ± 3.96 ^ab^
FJH6	18.80 ± 0.66 ^c^	0.36 ± 0.04 ^a^	0.14 ± 0.02 ^a^	0.16 ± 0.01 ^b^	82.15 ± 0.77 ^a^	338.31 ± 3.77 ^a^
Valea Vinului area	18.12 ± 0.70 ^A^	0.19 ± 0.00 ^A^	0.08 ± 0.01 ^A^	0.14 ± 0.01 ^A^	81.38 ± 0.74 ^A^	335.43 ± 2.80 ^A^
FJH7	19.33 ± 1.06 ^b^	0.3 ± 0.08 ^a^	0.03 ± 0.04 ^c^	0.12 ± 0.01 ^b^	80.2 ± 1.00 ^b^	330.46 ± 4.40 ^b^
FJH8	18.60 ± 0.91 ^c^	0.21 ± 0.02 ^b^	0.06 ± 0.03 ^b^	0.12 ± 0.01 ^b^	78.94 ± 1.03 ^c^	324.79 ± 6.13 ^bc^
FJH9	20.70 ± 1.12 ^a^	0.38 ± 0.07 ^a^	0.12 ± 0.00 ^a^	0.10 ± 0.01 ^b^	80.93 ± 0.96 ^b^	333.46 ± 5.43 ^a^
Bocsig area	19.54 ± 1.06 ^A^	0.29 ± 0.008 ^A^	0.07 ± 0.04 ^A^	0.11 ± 0.01 ^B^	80.02 ± 1.00 ^A^	329.57 ± 4.40 ^A^

Different superscript lowercase letters within each row indicate statistically significant differences between the samples at a confidence level of *p* < 0.05. Different superscript uppercase letters denote significant differences among the areas of collection. Reported results represent the mean ± standard deviation values obtained from three replicates (*n* = 3).

**Table 4:** The phenolic content and antioxidant capacity of FJH samples from three different geographical areas of Romania.

Parameter	FJH1	FJH2	FJH3	Merișor Area	FJH4	FJH5	FJH6	Valea Vinului Area	FJH7	FJH8	FJH9	Bocsig Area
TPC (mg GAE/100 g)	90.12 ± 0.10 ^d^	90.23 ± 0.95 ^d^	99.79 ± 1.01 ^c^	93.38 ± 5.55 ^A^	120.08 ± 0.62 ^a^	110.24 ± 0.46 ^b^	110.71 ± 0.85 ^bc^	113.67 ± 25.55 ^A^	89.87 ± 0.95 ^d^	89.92 ± 0.79 ^d^	119.88 ± 0.82 ^a^	99.89 ± 17.31 ^A^
TFC (mg CE/100 g)	19.71 ± 1.23 ^e^	18.13 ± 0.22 ^f^	23.13 ± 0.20 ^d^	20.32 ± 2.55 ^A^	33.75 ± 0.73 b	26.25 ± 0.40 ^c^	39.38 ± 0.49 ^a^	33.12 ± 6.58 ^A^	18.75 ± 0.38 ^ef^	20.17 ± 0.47 ^e^	29.78 ± 0.98 ^c^	22.90 ± 6.00 ^A^
DPPH (%)	54.00 ± 2.12 ^ab^	55.87 ± 0.36 ^a^	53.40 ± 2.36 ^b^	54.42 ± 1.28 ^A^	44.09 ± 0.26 ^c^	43.16 ± 0.32 ^c^	40.09 ± 0.42 ^d^	42.44 ± 2.09 ^B^	39.31 ± 0.20 ^d^	37.69 ± 0.80 ^e^	35.41 ± 0.15 ^f^	37.46 ± 1.95 ^C^
IC_50_	9.9.44 ± 0.35 ^d^	8.88 ± 0.10 ^e^	7.83 ± 0.18 ^f^	8.71 ± 0.81 ^B^	11.30 ± 0.09 ^c^	11.49 ± 0.11 ^c^	12.54 ± 0.13 ^b^	12.53 ± 0.72 ^A^	12.74 ± 0.07 ^b^	13.27 ± 0.28 ^ab^	14.15 ± 0.06 ^a^	13.38 ± 0.71 ^A^
FRAP (mM Fe^2+^/100 g honey)	1.88 ± 0.07 ^b^	2.32 ± 0.10 ^a^	1.91 ± 0.08 ^b^	2.03 ± 0.24 ^A^	1.78 ± 0.06 ^c^	1.09 ± 0.09 ^e^	0.97 ± 0.10 ^f^	1.28 ± 0.43 ^B^	1.07 ± 0.05 ^e^	1.00 ± 0.02 ^f^	1.28 ± 0.09 ^d^	1.11 ± 0.14 ^B^

Data are presented as mean ± standard error (*n* = 3). Different superscript letters in the same row represent significant differences between the accumulation of phenolic compounds and antioxidant capacity of the samples. Different superscript uppercase letters denote significant differences according to the regions of collection.

## Data Availability

The original contributions presented in the study are included in the article/Supplementary Materials, further inquiries can be directed to the corresponding author.

## Supplementary Materials

The following supporting information can be downloaded at: https://www.mdpi.com/article/10.3390/foods13131959/s1, Supplementary Table S1: Family and plant species of the pollen types from the analyzed honey samples; Supplementary Table S2. Importance of variables and components of computed PCs.
