# Supplementary material for: Unraveling the Physicochemical, Nutritional and Antioxidant Properties of the Honey Produced from the Fallopia japonica Plant
Source: Foods. 2024 Jun 21;13(13):1959. doi: 10.3390/foods13131959 (PMC11240986; doi:10.3390/foods13131959)
Supplement: Supplementary file 1 [file foods-13-01959-s001.zip › foods-3053890-supplementary.pdf]

Supplementary Table S1. Family and plant species of the pollen types from the analyzed honey samples.

**Samples**	**Predomminant pollen (>45%) Family-Species**	**Secondary pollen (16- 45%) Family-Species**	**Important minor pollen (3-15%) Family-Species**	**Minor pollen (<3%) Family-Species**
**Merisor area-Maramureș County**		**Fagaceae**<br>*Castanea sativa*<br>**Asteraceae**<br>*Helianthus annuus*	**Polygonaceae**<br>*Fallopia japonica*<br>**Brassicaceae**<br>*Brassica sp.*<br>**Asteraceae**<br>*Centaurea jacea type*	**Asteraceae**<br>*Matricaria type*<br>**Fabaceae**<br>*Trifolium sp.*<br>**Boraginaceae**<br>*Echium sp.*<br>*Symphytum sp.*<br>**Gramineae**<br>*Zea mays*<br>**Asteraceae**<br>*Ambrosia sp.*<br>**Rhamnacea**<br>*Rhamnus sp.*<br>**Balsaminaceae**<br>*Impatiens sp.*<br>**Rosaceae**<br>*Filipendula sp.*
**Valea Vinului area-Satu Mare Country**		**Fabaceae**<br>*Trifolium sp.*<br>**Asteraceae**<br>*Helianthus annuus*	**Polygonaceae**<br>*Fallopia japonica*<br>**Asteraceae**<br>*Centaurea jacea type*<br>**Rosaceae**	**Asteraceae**<br>*Cirsium-type*<br>**Asteraceae**<br>*Matricaria type*<br>**Asteraceae**<br>*Ambrosia sp.*<br>**Fabaceae**<br>*Coronilla type*<br>**Boraginaceae**<br>*Echium sp.*<br>**Asteraceae**<br>*Taraxacum sp.*
**Bogsig area**		**Rosaceae**	**Polygonaceae**	**Fabaceae**

**Arad country**	**Brassicaceae**<br>*Brassica sp.*<br>**Malvaceae**<br>*Tilia sp.*	*Fallopia japonica*<br>**Asteraceae**<br>*Centaurea jacea type*	*Trifolium sp.*<br>**Asteraceae**<br>*Taraxacum sp.*<br>**Asteraceae**<br>*Helianthus annuus*<br>**Asteraceae**<br>*Matricaria type*<br>**Lamiaceae**<br>**Boraginaceae**<br>*Echium sp*<br>**Cornaceae**<br>*Cornus sp.*

Supplementary Table S2. Supplementary files of computed PCs.

Importance of variables

**PC1**		**PC2**		**PC3**	
DPPH (%)	-0.43176	DPPH (%)	-0.1845499	DPPH (%)	0.18190877
IC$_{50}$	0.423933	IC50	0.236708	IC50	-0.18115389
FRAP(mM Fe^{2+}/100 g honey)	-0.36904	FRAP(mM Fe2+/100 g honey)	-0.1903412	FRAP(mM Fe2+/100 g honey	0.19187449
Water content (%)	-0.08795	Water content (%)	-0.299802	Water content (%)	-0.75790772
Proteins (%)	0.426716	Proteins (%)	-0.1608727	Proteins (%)	0.06587293
Lipids (%)	0.16181	Lipids (%)	-0.5051641	Lipids (%)	-0.3369015
Salt (g/L)	-0.28558	Salt (g/L)	-0.4337285	Salt (g/L)	0.05316045
TPC (mg GAE/100 g)	0.314806	TPC (mg GAE/100 g)	-0.3616743	TPC (mg GAE/100 g)	0.32476355
TFC (mg CE/100 g)	0.317826	TFC (mg CE/100 g)	-0.4287192	TFC (mg CE/100 g)	0.31098759
**PC4**		**PC5**		**PC6**	
DPPH (%)	0.049755	DPPH (%)	-0.23245192	DPPH (%)	0.197998825
IC$_{50}$	0.016152	IC50	-0.0908296	IC50	0.097666809
FRAP(mM Fe^{2+}/100 g honey)	0.56885	FRAP(mM Fe2+/100 g honey)	-0.26788412	FRAP(mM Fe2+/100 g honey)	0.219812455
Water content (%)	0.391189	Water content (%)	0.32477394	Water content (%)	-0.000618273
Proteins (%)	0.223672	Proteins (%)	0.15470305	Proteins (%)	0.619268537

Lipids (%)	-0.30733	Lipids (%)	-0.67758134	Lipids (%)	-0.080029483
Salt (g/L)	-0.4577	Salt (g/L)	0.51066516	Salt (g/L)	0.01738577
TPC (mg GAE/100 g)	0.388591	TPC (mg GAE/100 g)	0.10110513	TPC (mg GAE/100 g)	-0.661569547
TFC (mg CE/100 g)	-0.12519	TFC (mg CE/100 g)	0.08015867	TFC (mg CE/100 g)	0.273979912
**PC7**		**PC8**		**PC9**	
DPPH (%)	-0.30557	DPPH (%)	-0.74138452	DPPH (%)	-0.087728
IC_{50}	0.410363	IC50	-0.52514338	IC50	0.5188544
FRAP(mM Fe^{2+}/100 g honey)	0.400589	FRAP(mM Fe2+/100 g honey)	0.2869281	FRAP(mM Fe2+/100 g honey)	0.3229513
Water content (%)	0.072287	Water content (%)	-0.14070931	Water content (%)	-0.2107794
Proteins (%)	-0.52305	Proteins (%)	0.13170869	Proteins (%)	0.1982754
Lipids (%)	-0.10083	Lipids (%)	0.14720544	Lipids (%)	0.1153804
Salt (g/L)	0.075652	Salt (g/L)	0.01066114	Salt (g/L)	0.501094
TPC (mg GAE/100 g)	-0.1636	TPC (mg GAE/100 g)	-0.14441535	TPC (mg GAE/100 g)	0.1345302
TFC (mg CE/100 g)	0.506263	TFC (mg CE/100 g)	-0.11162661	TFC (mg CE/100 g)	-0.5025426

Importance of components

**PC1**		**PC2**		**PC3**	
Standard deviation	2.1451	Standard deviation	1.4511	Standard deviation	1.0542
Proportion of Variance	0.5113	Proportion of Variance	0.234	Proportion of Variance	0.1235
Cumulative Proportion	0.5113	Cumulative Proportion	0.7452	Cumulative Proportion	0.8687
**PC4**		**PC5**		**PC6**	
Standard deviation	0.8137	Standard deviation	0.57517	Standard deviation	0.32325
Proportion of Variance	0.07357	Proportion of Variance	0.03676	Proportion of Variance	0.01161
Cumulative Proportion	0.9423	Cumulative Proportion	0.97905	Cumulative Proportion	0.99066
**PC7**		**PC8**		**PC9**	
Standard deviation	0.28118	Standard deviation	0.07044	Standard deviation	4.77E-18
Proportion of Variance	0.00878	Proportion of Variance	0.00055	Proportion of Variance	0.00E+00
Cumulative Proportion	0.99945	Cumulative Proportion	1	Cumulative Proportion	1.00E+00
